# Identifying and Understanding the Health Information Experiences and Preferences of Caregivers of Individuals With Either Traumatic Brain Injury, Spinal Cord Injury, or Burn Injury: A Qualitative Investigation

**DOI:** 10.2196/jmir.7027

**Published:** 2017-05-10

**Authors:** Nathan T Coffey, James Cassese, Xinsheng Cai, Steven Garfinkel, Drasti Patel, Rebecca Jones, Dahlia Shaewitz, Ali A Weinstein

**Affiliations:** ^1^ Center for the Study of Chronic Illness and Disability George Mason University Fairfax, VA United States; ^2^ American Institutes for Research Washington, DC United States

**Keywords:** traumatic brain injury, burns, spinal cord injuries, caregivers, health information, consumer, qualitative research

## Abstract

**Background:**

In order to meet the challenges of caring for an injured person, caregivers need access to health information. However, caregivers often feel that they lack adequate information. Previous studies of caregivers have primarily focused on either their time and emotional burdens or their health outcomes, but the information needs of caregivers have not been thoroughly investigated.

**Objective:**

The purpose of this investigation was to identify the preferred sources of health information for caregivers supporting individuals with injuries and to explore how access to this information could be improved.

**Methods:**

A total of 32 caregivers participated in semistructured interviews, which were used in order to develop a more in-depth understanding of these caregivers’ information needs. Digital audio recordings of the interviews were used for analysis purposes. These audio recordings were analyzed using a thematic analysis or qualitative content analysis. All of participant’s interviews were then coded using the qualitative analysis program, Nvivo 10 for Mac (QSR International).

**Results:**

The caregivers endorsed similar behaviors and preferences when seeking and accessing health information. Medical professionals were the preferred source of information, while ease of access made the Internet the most common avenue to obtain information. The challenges faced by participants were frequently a result of limited support. In describing an ideal health system, participants expressed interest in a comprehensive care website offering support network resources, instructive services about the injury and caregiving, and injury-specific materials.

**Conclusions:**

According to the participants, an ideal health information system would include a comprehensive care website that offered supportive network resources, instructive services about the injury and caregiving, and materials specific to the type of patient injury.

## Introduction

More than 52 million US residents act as a caregiver to an adult aged 18 years or older [1]. That number increases to over 65.7 million for the US adult population that care for patients of all age groups [2]. Almost two-thirds of those receiving care have a long-term physical condition [3]. The caregivers supporting these individuals are commonly unpaid persons, including family, friends, and neighbors of the injured or ill [2]. For this investigation, participants are self-identified as the primary caregiver, either in a full-time or part-time capacity.

Caregivers assume responsibilities that affect their personal lives, financial status, and mental well-being [4]. They often provide housing, coordinate rehabilitative services, and communicate with health care providers [5]. An injury, by its very nature, is the result of an acute event. Unlike chronic conditions that can be slow moving, those caring for an injured person are thrust into the position with no time to prepare. Previous examinations of those providing care to individuals with traumatic brain injury (TBI) found that they frequently feel overwhelmed [6,7]. Low health-related quality of life among TBI caregivers has been associated with decreased mental health status [8]. Caregivers of individuals with spinal cord injury (SCI) can experience depressive symptoms at similar rates as the injured person [9], whereas caregivers of individuals with burn injuries experience more pronounced symptoms of depression than caregivers of individuals who are hospitalized for other reasons [10].

Many caregivers experience unmet support needs, including insufficient care-related information, unsatisfactory emotional support, and difficulty maintaining relationships [11-15]. The majority of caregivers lack the time or energy to care for themselves and often experience declining health after assuming that responsibility [6,16]. Among parents caring for children with a burn injury, perceived lack of support was significantly associated with symptoms of general anxiety and depression [15].

In order to meet the challenges of caring for an injured person, caregivers need access to health information. However, caregivers often feel that they lack adequate information and support [11,14]. Previous examination of TBI caregivers found that health information was one of their most important needs [12]. Caregivers also seek methods of accessing services and support [14]. Information about managing physical and emotional stress is highly sought as well [17].

Health information is critical for caregivers to provide the most effective care for the patients [5]. Family caregivers commonly perform medical or nursing tasks [18]. Consequently, caregivers seek Web-based reviews of particular drugs or medical treatments more frequently than noncaregivers [19]. A lack of information can lead to frustration and anxiety about the short- and long-term effects of the injury [4]. Common sources of health information for caregivers include medical professionals, written sources, family or friends, nonprint media, and the Internet [20]. Those with higher health literacy are more likely to obtain information from multiple sources [20]. However, many potentially beneficial activities, including independent research regarding care, may be hindered due to unmet support needs such as managing concerns and reducing stress [21].

TBI, SCI, and burn injuries are prevalent throughout the United States and are severe in nature [22-24]. According to the Centers for Disease Control and Prevention (CDC), over 2 million patients were admitted to the hospital for a TBI in 2010 [22]. Another 40,000 were hospitalized for a burn injury [24]. There are about 12,500 new cases of SCI every year according to the National Spinal Cord Injury Statistical Center (NSCISC) [23]. Due to their severity, all three injuries can require long-term care and rehabilitation; thus the need for health information among caregivers for persons with these injuries is pronounced. Previous studies of caregivers have primarily focused on either their burdens or health outcomes. Additionally, many prior studies focused on the challenges associated with either pediatric or elder care. This qualitative study seeks to gain insight into the preferred sources of health information for caregivers supporting individuals with TBI, SCI, or burn injuries to ensure a greater depth of understanding in this area than in previous work. Additionally, the investigators sought to assess the obstacles faced by those caring for persons with these injuries when accessing services and information through the health care system, and how access to these items could be improved.

## Methods

### Subject Recruitment and Selection

Subjects were recruited through a number of different outreach methods. First, National Institute on Disability, Independent Living, and Rehabilitation Research (NIDILRR)–funded SCI, TBI, and Burn Model Systems grantees were contacted to recruit participants. The grantees were asked to identify potential participants and direct candidates to the recruitment website. Subjects were also solicited through advertisements placed in printed materials, websites, as well as social media sites. Finally, participants were reached by sending recruitment emails provided by TBI, SCI, and burn consumer advocacy groups.

Subjects were selected to participate if they self-identified as the primary caregiver of a person with either a TBI, SCI, or burn injury. The number of hours per week spent on caregiving was not an inclusion/exclusion criterion. All caregivers needed to be at least 18 years of age and could care for a recipient of any age. Informed consent was obtained (orally) from all of the participants and assurances of anonymity/confidentiality given. Participant distress and safeguarding protocols were established. The Institutional Review Boards of both the American Institutes of Research and George Mason University approved this investigation.

### Data Collection

Semistructured interviews were used in order to develop a more in-depth understanding of caregivers’ information needs. A psychologist (AAW) trained all interviewers on the proper techniques in conducting semistructured interviews. These trained interviewers were an occupational therapist and Masters of Public Health graduate students. The interview consisted of three sections. In the first section of the interview, caregivers were asked to discuss the subject’s care background as well as the activities of daily living and instrumental activities of daily living of the person receiving care. The second section used a questionnaire to assess the various types of needs caregivers may have. The final open-ended section of the interview examined the individual’s medical, rehabilitation, and continuing care experience, specifically focusing on information needs assessment (Textbox 1). For this qualitative investigation, only the open-ended portion of the interviews is reported. The investigators used a semistructured interview guide that allowed consistency in questions that were asked across interviews. The interviews were conducted and recorded over the phone for participants’ convenience and to allow for geographic variability in the location of the caregivers’ residences.

**Open-ended question prompts.**

- Thinking back over the time since the injury, what has been most difficult for you?
- What has been the most helpful for you since the injury?
- We had discussed your health earlier in the interview, and I was wondering if you feel that your health has changed since becoming a caregiver?
- Now, we are going to spend some time focusing on the informational component of your life as a caregiver. So, how do you currently receive information related to (TBI, SCI, and burn injury)?
- When you do receive or access information, how, if at all, do you use the information?
- Has the information that you received or accessed been useful? Pertinent to your needs? Accurate? Understandable? Trustworthy?
- What are the biggest difficulties you face in accessing the kind of information you want about the patient’s (or loved one if appropriate) condition?
- I want you to imagine that we can start from scratch and develop a new approach for getting information to caregivers. In your dream world, what would this new model look like? Don’t worry about the money required, or whether your idea is logistically feasible.

### Analysis

Digital audio recordings of the interviews were used for analysis purposes. These audio recordings were analyzed using the framework approach [25]. This approach is within the broad family of analysis methods often termed “thematic analysis” or “qualitative content analysis.” This type of analysis was used to draw descriptive or explanatory conclusions clustered around themes, while allowing the ability to analyze the data across individuals as well as within individuals. The research team, before setting up a thematic structure, familiarized themselves with the interviews [26] by listening to the recordings and reviewing notes taken by the interviewers during the interviews. Then, the analysis team listened to a random selection of interviews that included all injury types (TBI, SCI, and burn) to create a coding system. These researchers then met to discuss the coding and establish a set of codes that would be applied to all of the interviews. Once the coding system had been reviewed and refined, the two researchers who would code all of the interviews, coded the same interview to determine interrater reliability (IRR) and the systematic application of codes. Nvivo’s coding comparison query showed a 96.4% agreement (kappa coefficient, κ=.65). Percentage agreement refers to the percentage where the two users coded the data in the same way. Since audio files were coded, the unit of measurement is duration in seconds. Average percentage agreement and kappa values indicated consistent interpretation of codes by the different interview coders.

All of participant’s interviews were then coded using Nvivo. In some cases, a participant would respond to a question in a way that fell beyond the scope of the developed coding system. In those cases, a new code was added and the remaining research team members were notified of the change. In total, 5 codes were modified during the coding process.

The coding system was then used to develop an analytical framework. The codes were grouped together into categories. In order to facilitate this process, the tree map feature was used to visually represent potential categories. The researchers then defined these groupings of codes. The last step was the interpretation of the data. The researchers met many times to discuss the characteristics of and differences between the data.

## Results

### Participant Characteristics

A total of 40 caregivers provided consent and were interviewed. However, 8 of the interview audio files were unable to be read by the Nvivo program. Thus, the analysis was based on the remaining 32 caregiver interviews, of which 16 providing care to a person with a TBI, 10 providing care to a person with a SCI, and 6 providing care to a person with a burn injury. The open-ended response section of interviews lasted 34 min on average, ranging from 12 min to 115 min.

Study participants had provided care for 7.0 years (SD 8.4) on average. Details of the study population’s ethnicity, socioeconomic status, and injury type can be found in Table 1.

**Table 1:** Demographics of the care recipients and the caregivers.

Characteristics of the sample		Total (N=32)	TBI^a^ (n=16)	SCI^b^ (n=10)	Burn (n=6)
**Recipient age^c^ (in years)**				
	Under 18	3	1	1	1
	18-29	10	6	2	2
	30-39	3	3	0	0
	40-49	5	1	3	1
	50 and over	10	4	4	2
**Recipient gender**				
	Male	27	14	9	4
	Female	5	2	1	2
**Recipient insurance status**				
	Insured	29	14	10	5
	Uninsured	3	2	0	1
**Caregiver age^d,e^ (in years)**				
	18-29	0	0	0	0
	30-39	6	2	2	2
	40-49	10	4	3	3
	50 and over	13	8	4	1
**Caregiver gender**				
	Male	2	1	0	1
	Female	30	15	10	5
**Caregiver education level^f^**				
	High school	7	3	2	2
	Associates degree	2	1	0	1
	1-3 years of college	10	6	4	0
	Bachelor’s degree	7	3	1	3
	Graduate school	5	2	3	0
Length of time caring for patient (in years), mean (SD^g^)		7.0 (8.4)	7.1 (6.7)	10.1 (11.7)	1.5 (1.5)
Hours per week spent caring for patient, mean (SD)		67.9 (59.1)	73.6 (64.2)	62.3 (53.0)	61.8 (63.6)

^a^TBI: traumatic brain injury.

^b^SCI: spinal cord injury.

^c^For TBI, n=15.

^d^For TBI, n=14.

^e^For SCI, n=9.

^f^For TBI, n=15.

^g^SD: standard deviation.

### Preferred Sources of Health Information

The majority of subjects (n=30) received injury-related information via the Internet. The most common ways caregivers accessed Web-based information was through sites found using search engines (n=20) and medical websites (n=20). Not all types of Internet resources were widely used; a smaller proportion of caregivers visited government (n=10), support group (n=9), or discussion board (n=5) websites regularly.

### Health Information Access and Use

#### Interference With Accessing Information

Caregivers described difficulties that interfered with their ability to access information about treatment, caregiving, and health conditions. When asked to describe their greatest obstacle to accessing information, caregivers most commonly cited challenges simply finding information (n=10), followed by medical/insurance bureaucracy (n=9), unsupportive medical staff (n=7), and lack of support from sources other than medical staff (n=4). The majority of caregivers (n=28) believed that “access to the Internet” did not hinder their ability to access health information. Similarly, difficulty in finding “culturally appropriate” information did not hinder access (n=26). In contrast, caregivers believed that the “accessibility to information (other than the Internet)” (n=15), “time to search” (n=14), the “understandability of information” (n=12), and obtaining “credible information” (n=10) at least partially interfered with their ability to access to injury-related information. Only 8 caregivers indicated that they did not face any difficulties when accessing information. However, finding concrete information still proved to be a challenging task due to the inaccessibility of specialized care or gaps in medical research.

There are just so few people that are knowledgeable in our area, and probably in most geographic areas, about the specialized needs of people with spinal cord injury; especially quadriplegics. There are so many systems of the body affected and there aren’t too many (specialists) in any one given area who really understand all the ramifications of how the injury’s affecting all the body systems.

Interview 2003; SCI

I think just finding (health information) was the biggest challenge. There’s not a lot known about burns, relatively speaking. There’s a lot of anecdotal stuff from survivors but there’s not a lot publishedresearch on burns and burn recovery.

Interview 1002; burn

We’ve chosen doctors who are, who will listen. Who are open to, if they don’t know the answer, taking what we say, and checking it. But last time we looked for a doctor, I actually went and interviewed doctors...because there aren’t a lot of people that know about (patient’s) exact condition.

Interview 2011; SCI

#### Information-Seeking Behaviors

Themes surrounding the process of gaining health information emerged seeking different types of information, utilizing multiple sources, and making comparisons. The process of locating information involved extensive preparation, persistence, and due diligence.

Although there were differences in the types of health information individual caregivers sought, common themes emerged. Caregivers researched information that was directly related to supporting the individual receiving care. “Treatment” (n=14), “rehabilitation” (n=13), and “medication” (n=11) emerged as dominant themes. Due to the inherent chronicity of these serious injuries, many caregivers sought information on “long-term care” (n=15), that is, research on the long-term effects of patient conditions and sequela, and adjustments to disabilities.

For caregivers, an important source of health information was seeking information through individuals who had similar experiences. These opportunities for seeking information could be from various outlets including organized support groups designed to share experiences with those in similar situations, informal sharing, development of coping strategies with professional counselors, doctor relationships, or rehabilitation settings with clinical specialists and organizations centered on TBI, SCI, or burn survivors.

When I have actually sought counseling...about... trying to adapt and cope with this kind of new person that I’m dealing with. That’s been very helpful to me to have some support person that I can call.

Interview 2009; SCI

we call it the working support group, because it was headed by a neuropsychologist, and he would actually have the survivors worktrying to improve themselves. So that has been very useful. The support group that we go to here in our town is more of a social kind of thing

Interview 3015; TBI

Caregivers utilized a number of different sources to acquire health information. Common sources included “Web-based research” (n=26), “doctor instructions” (n=16), “medical journals” (n=14), and “injury organizations” (n=13). Web-based communication, library visits, clinical trials, and other persons with similar injury experiences were referenced, albeit less frequently. Caregivers often compared information from multiple sources (n=21) to evaluate its quality and corroborate its accuracy.

I spent a lot of time, really the first 4 monthsdoing PubMed searches, and reading literature, and reading textbooks on burns, and all that kind of stuff. That was really the first thing I did, was kind of educate myself about burn and the burn process and recovery and healing.

Interview 1008; burn

Everything and anything I could get my hands on to understand (the injury). I have visited WebMD, the traumatic brain injury model centers...the National Traumatic Brain Injury Association. I’ve been out there on all the websites.

Interview 3001; TBI

#### Information Utility

Personal experience dealing with an injury also served as a reference point for caregivers. Caregivers retrospectively compared their patient’s outcomes with Web-based information they received, enabling them to determine its usefulness, accuracy, and understandability. However, the caregivers faced a steep learning curve when acclimating themselves with injury-related health information early in their transition to a caregiver role.

When I did read about the burns and different things like that, we may not have been at the stage that it talked about, but we did get there. And I could remember times where it was like “oh, that is what they said was going to happen.”

Interview 1004; burn

I did understand it, but after, going, advocating and learning more, and then going back to some of the older documents that I had researched, and rereading it, I understood it more, because I guess I had actually experienced some of it.

Interview 3001; TBI

Knowledge of various treatment options also influences caregiver actions and their respective patient’s rehabilitative outcomes. Caregivers reported instances of reliance on their own judgment based on independent research. This sometimes led to negotiation or collaboration with medical professionals.

...there’s very little research on the subject, but there’s some, about the link between things like fish oil and brain injury. And, I had to try that, and noticed a really big improvement in both areas, in terms of clarity of thinking and his skin condition. It’s anecdotal, I have no proof, but I really feel like it’s made a difference. It’s not something I would have considered without looking at websites.

Interview 3003; TBI

a few years ago I said to his physician, shouldn’t he be tested for osteoporosisI had learned online that quads are at high-risk for osteoporosis. So he agreed to test him, and did test him, and indeed he did have osteoporosis.

Interview 2011; SCI

If we’re talking about medications or treatments, or, you know things that I may have read about but no one has suggested. So I kind of throw that out there like neurofeedback, you know, as something that I ran across, you know on the Internet, and benefits for survivors of traumatic brain injury. And so I brought that up to one of his neuropsychologists and we have adopted...getting him involved with neurofeedback.

Interview 3017; TBI

### Improvements to Health Information Access

The final open-ended interview question asked participants to describe their ideal system for getting health information to caregivers without having to consider practical limitations such as cost. In doing so, respondents offered a variety of potential modifications to health information systems that they felt would be beneficial to caregivers. In conceptualizing model systems, participants often focused on health information, support services, and modes of delivery most applicable to them. As a result, responses diverged from one another, but maintained commonalities in information sought, types of services, and access to information.

#### Health Information

Support network information (n=26) was the most common response, though again employing a multitudinous definition of support, including organized support groups, information for and from people with shared experiences, resources to access services related to rehabilitation, ongoing care, and daily living.

I would want the information probably right after whatever had happened and I wouldwant some positive reinforcement, and just general information, and maybe somesupport right off the bat from other people; other people that have gone through thiswho have experienced the same thing. And it needs to be positive interaction, not negative.

Interview 3009; TBI

Caregivers also expressed a desire for access to updated publications (n=11) as a method of staying well-informed with current research. Guides covering treatment, recovery, and rehabilitation, via the Internet (n=5) or in hard-copy form (n=9), were suggested as well. Those guides would serve as an educational tool to help prepare caregivers and thus mitigate their risk of feeling overwhelmed following the injury event.

I think it would be great to have a manual on burn injury and recovery that is categorized by subject matter and is in both print and online form and easily available to all patients and family members. That has put together in one place, all known and accurate current research and has weeded out anything that has either old and has been proven inaccurate, or irrelevant, or whatever. So it’s done all the librarian work for us, so that we have it all in one place.

Interview 1002; burn

There’s no good, central clearinghouse. It’d be great if there was one place on the Web you could go that had links to all these places. That had the government links, that had care-cure forums, that had Avery biomedical, every place that, that can help you. Every place that can help you, like with durable medical supplies, a place that can help you with...hooking up with caregivers. Places that could help you connect with other people in your situation.

Interview 2011; SCI

#### Support Services

As respondents discussed the services that they believed should be available in a model health system, two themes emerged. The first was caregiver support services. Specifically, resources such as “support groups” (n=22), “care coordinators” (n=15), “caregiver training” (n=14), and “classes on injury condition, treatment, and life” (n=14) were frequently suggested to prepare caregivers and their families for the rigors of caring for someone with TBI, SCI, or burn.

Everybody needs to be professionally trainedwhether it be education, legal, medical, or whatever, that the proper people are trained and are able to explain, or tell the caregivers and the survivor, what to expect, where they can go, and so forth. So I think the training is very much needed.

Interview 3001; TBI

Several respondents recommended support group sessions with people that had similar experiences for patients and their caregivers, while they are still in the hospital.

A support group led by a professional, but run by, and taken over by the people who are the caregivers, or the family membersthe one paid person would actually get the real information and give it to the family members, and the family members in turn could be providing things that they learned, which in turn could be coordinated and disseminated as information.

Interview 2010; SCI

I wish there was somebody like myselfthat could come into a burn survivor and their family’s room and make that connection and answer their personal questionsif I would have had somebody like me or my other SOAR (Survivors Offering Assistance in Recovery) volunteer members come into my room, even that first week, I feel like my life would have been so much easier. I felt so lost.

Interview 3016; burn

The second theme that emerged was medical care services. Respondents expressed their desire for “rehabilitation services” (n=19), “medical specialists” (n=13), and “financial services” (n=6). In many cases, respondents expressed their desire for their local health care provider to recreate the services offered at state-of-the-art facilities. Physical access to specialized treatment facilities can be a substantial barrier; therefore, caregivers prioritized proximity and transportation to health care facilities.

#### Information Access

Caregivers most frequently recommended that an ideal health information system be accessed through in-person communication (n=20) or via the Internet (n=18). A combination of these two methods, such as a “livechat” feature (n=7), was cited to allow for interactive question and answer sessions. Printed materials (n=9), phone systems (n=8), and television programs (n=3) were also recommended as resources for sharing information or to serve as learning materials for caregivers. In the context of comprehensive Web-based information, the user-friendliness of the Web page and an effective search function emerged as critical features.

At least if you have that human, that person-to-person dialogue going, you can sometimes get answers to things that are specific to your own situation, instead of reading through a Q&A or something that’s posted.

Interview 1004; burn

The Internet, of course, is the easiest and fastest way for me to get information besides talking to actual doctors in the hospital.

Interview 3001; TBI

It would be nice if there was a hotline you could call if you were in a situation where you needed somebody to talk to right away. There are a lot of urgent situations when you are dealing with disability and I wish there was some kind of caregiver hotline that would not only support you emotionally, but maybe help alleviate some of the stress when you feel like you’re dealing with something completely alone. [Interview 2003; SCI]

Interview 2003; SCI

## Discussion

### Principal Findings

This study offers insights into the challenges faced by individuals caring for persons with TBI, SCI, and burn injuries. Participants revealed the obstacles that they faced, including gaps in formal and informal support systems, emotional strain, and a limited ability to access health services and health information resources. The interviews further revealed patterns and preferences for seeking health information among those caring for individuals with TBI, SCI, and burn injuries.

Although previous research has examined the burden experienced by individuals caring for elderly persons or degenerative conditions associated with aging, such as dementia, relatively few studies have compared the burden experienced by those caring for life-long injuries. This study finds that medical professionals are the preferred method of receiving health information among TBI, SCI, and burn caregivers, similar to the preference of those caring for other conditions [27]. Nevertheless, the Internet was an integral health information resource for caregivers of persons with all three injuries. It is important to remember that one of our recruitment techniques was Web-based, therefore, our results may be positively biased toward Internet resources. These findings suggest that caregivers of these injuries rely on the Internet in a similar capacity as those caring for persons with chronic or long-term diseases [17,20,28]. Furthermore, our study participants sought health information from multiple sources beyond the Internet, much like caregivers of persons with other conditions [20,29,30]. These findings indicate that the methods and preferences of accessing health information among caregivers are similar regardless of the conditions being cared for.

When TBI, SCI, and burn caregivers sought health information, it was commonly related to medical care for the injured person. Caregivers are often required to perform medical tasks despite rarely having clinical training [3,18]. This underscores caregiver’s need for increased access to medical personnel. It also highlights the need for formal caregiver training, a desire strongly expressed by caregivers of every injury. Caregiver training has been demonstrated to improve caregiver self-efficacy and stress management [31-33]. Comprehensive health care systems should therefore incorporate structured training programs to reduce caregiver burnout and dependence on medical personnel.

The obstacles and preferences that caregivers discussed indicate a pronounced need for access to Web-based resources that provide synthesized care information and access to support resources. Features that combine the utility of the Internet with the expertise of medical professionals, including care-support hotlines, have been shown to reduce caregiver burden and therefore may improve their ability to provide care [34]. Thus, use of technology to improve communication with clinicians and support organizations should be prioritized by health care systems. Internet-based support for caregiving has been a valued model for TBI patients [35-37]. In addition, training caregivers on how to evaluate health care information on the Internet could be an excellent way to improve the caregivers’ confidence in accessing quality information.

### Strengths and Limitations

The in-depth, semistructured interviews allowed each participant to provide thorough insights into the health information issues related to their experience. Common experiences emerged, denoting the transferability of responses. Still, there were limitations. This study focused on individual interviews. A focus group study involving caregivers of individuals with these injury types may provide additional information because of the group interaction that is inherent in focus groups. Given the retrospective nature of the questions, there is a risk of recall bias from participants. In addition, this study had a small sample size and an uneven distribution of caregivers for each injury type. Study participants were predominantly recruited using the Internet which may have biased our sample in terms of preferences for Internet resources. The number of caregivers for each injury type was different, impacting the relative weight of responses from each study participant. Due to technical limitations, eight of the interview audio files had to be excluded from analysis. Caregivers were not asked if they were the only individuals who were caring for the recipient, which could have impacted their information needs.

The codebook itself could have been simplified by combining codes. Interrater reliability measures, percentage agreement, and kappa coefficient, indicated that both coders applied codes consistently, most likely due to the shared creation of the codebook, and follow-ups on interpretation of each code. However, IRR was not 100%, therefore minor discrepancies in code placement may be assumed.

### Conclusions

The detailed interviews conducted with TBI, SCI, and burn caregivers revealed the methods and resources that were used to acquire health information. Medical professionals were the preferred source of information, while ease of access made the Internet the most common. The challenges faced by participants were frequently a result of limited professional and social support. In describing an ideal health system, study participants expressed interest in a comprehensive care website that offered support network resources, instructive services about the injury and caregiving, and materials specific to their injury. An ideal health information system should incorporate methods of communicating directly with health professionals.

## Abbreviations

CDC: Centers for Disease Control and Prevention
IRR: interrater reliability
NIDILRR: National Institute on Disability, Independent Living, and Rehabilitation Research
NSCISC: National Spinal Cord Injury Statistical Center
SCI: spinal cord injury
TBI: traumatic brain injury
